# Giant Sigmoid Lipoma With Necrosis Mimicking Colorectal Cancer and Intussusception: A Case Report

**DOI:** 10.7759/cureus.101944

**Published:** 2026-01-20

**Authors:** Eduardo Gil Hurtado, Javier Luna García, Ulises J Soto Calvario

**Affiliations:** 1 General and Minimally Invasive Surgery, Hospital Angeles Universidad, Mexico City, MEX; 2 Minimally Invasive General Surgery, Centro de Formación en Cirugía de Mínima Invasión, Mexico City, MEX; 3 Colorectal Surgery, Nuevo Hospital Civil de Guadalajara "Dr. Juan I. Menchaca", Guadalajara, MEX; 4 General Surgery, Hospital General de Especialidades "Dr. Javier Buenfil Osorio", Campeche, MEX

**Keywords:** colorectal intussusception, gastrointestinal obstruction, giant colonic lipoma, laparoscopic sigmoidectomy, minimally invasive surgery

## Abstract

We describe a case of a giant sigmoid colon lipoma with clinical and radiological features mimicking colorectal carcinoma, highlighting the diagnostic and therapeutic challenges associated with large submucosal colonic lesions.

The patient presented with symptoms of bowel obstruction and cross-sectional imaging findings that highly suggested malignancy. The patient's clinical presentation, imaging studies, colonoscopic findings, and histopathological results were reviewed.

The patient underwent colonoscopic evaluation, which revealed a bulky, ulcerated lesion, further increasing suspicion for malignancy; due to the size of the lesion and inability to exclude cancer preoperatively, a laparoscopic segmental colectomy was performed.

Histopathology examination confirmed a giant submucosal lipoma with compression-related necrosis and associated chronic colitis. Postoperative recovery was uneventful.

This case emphasizes the value of correlating and integrating clinical, endoscopic, and imaging findings when evaluating large colonic lesions. Recognition of giant colonic lipomas as a potential mimic of colorectal carcinoma is essential to guide appropriate management and may help avoid unnecessary extensive surgical intervention.

## Introduction

A colonic lipoma is a benign, non-epithelial tumor composed of mature adipose tissue arising within the wall of the colon, most commonly from the submucosal layer [1-3]. These lesions are typically solitary, although up to 9% may be multiple, and they most frequently occur in the ascending colon, followed by the transverse, descending, and sigmoid colon [4,5]. The mean size is generally 1-2 cm, but larger lesions (often termed "giant" if >4 cm) can occur and are more likely to be symptomatic [4,5].

When symptomatic, colonic lipomas may present with abdominal pain, altered bowel habits, bleeding, or, rarely, obstruction or intussusception [1,3,5]. Endoscopic and cross-sectional imaging findings are often characteristic; however, large lesions may present imaging and clinical features similar to colorectal malignancy, posing diagnostic and therapeutic challenges [2,4].

## Case presentation

Clinical case

A 45-year-old female patient with no significant past medical history presented to the outpatient clinic with a three-month history of abdominal pain, described as intermittent, colicky, of moderate intensity, located in the periumbilical region with radiation to the lower left quadrant, which improved partially with analgesics, accompanied by constipation and intermittent rectal bleeding. On physical examination, abdominal inspection revealed mild distention; on auscultation, bowel sounds were present with slight hyperactive sounds suggestive of obstruction. Percussion of the abdomen demonstrated a predominantly tympanic note, without areas of abnormal dullness. Palpation of the abdomen revealed mild tenderness localized to the left lower quadrant with no palpable abdominal masses identified. There were no signs of peritoneal irritation.

**Figure 1:**
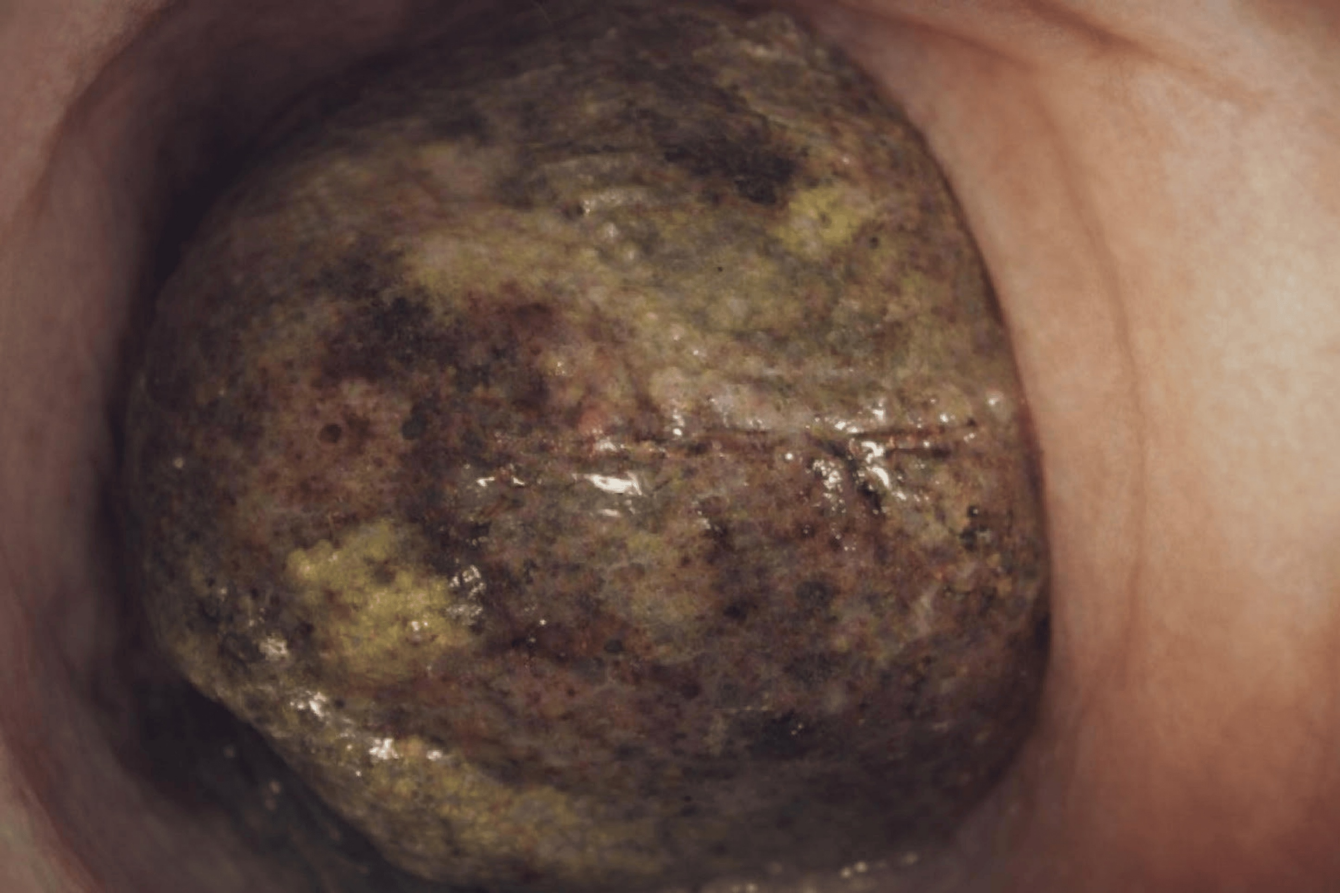
Intraluminal mass of approximately 10 cm of ovoid shape, which obstructs the entire lumen and prevents the passage of the endoscope

**Figure 2:**
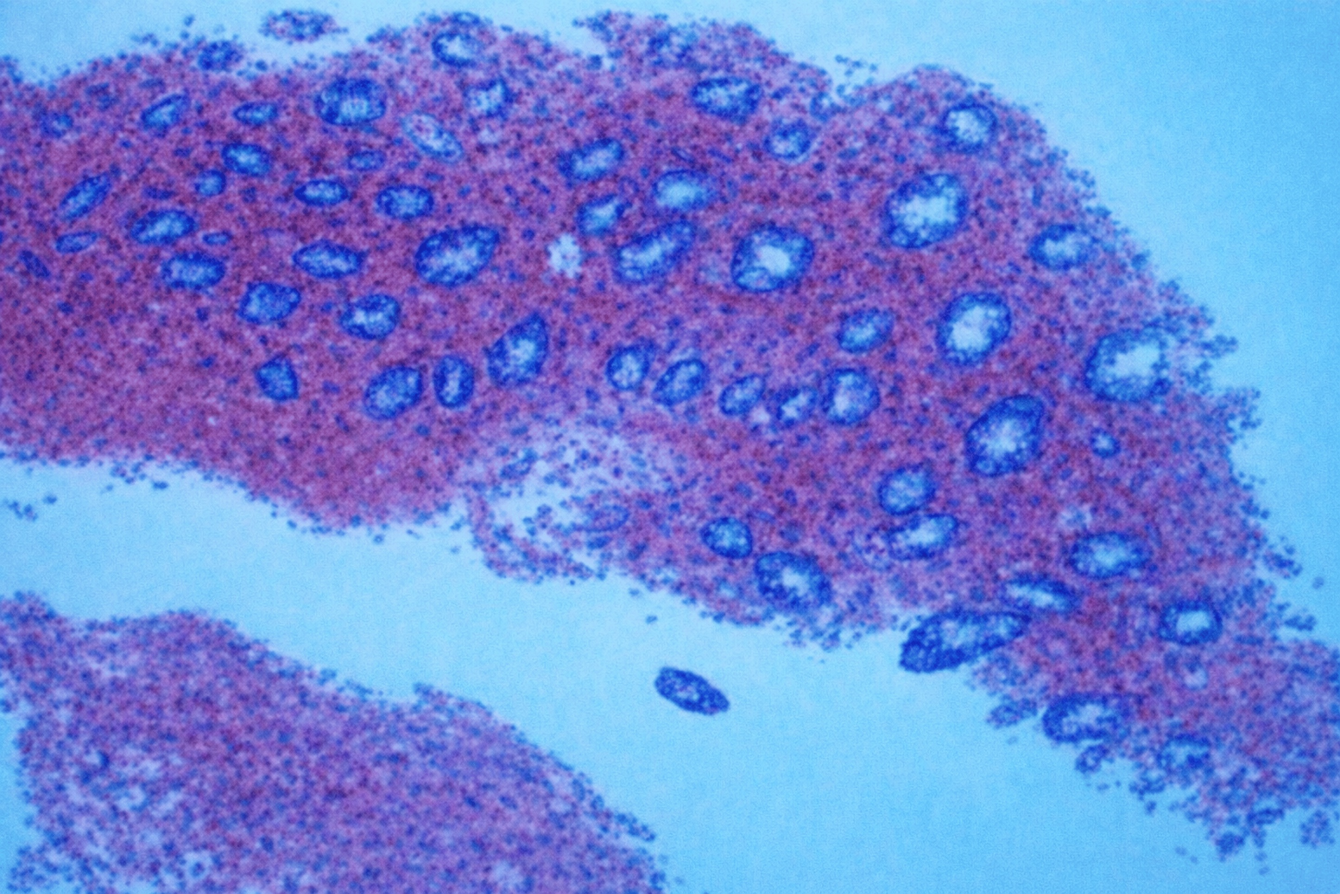
Moderate chronic irritative changes of the colonic mucosa and stromal hemorrhage and fragments of tissue with necrosis and hemorrhage, without evidence of neoplastic cells

Colonoscopy was performed and found a large intraluminal mass of approximately 10 cm in its greatest diameter of ovoid shape (Figure 1). The lesion occupied nearly the entire colonic lumen, beyond which the scope could not be passed, with the histopathological report indicating "moderate chronic irritative changes of the colonic mucosa and stromal hemorrhage and fragments of tissue with necrosis and hemorrhage, without evidence of neoplastic cells" (Figure 2).

**Figure 3:**
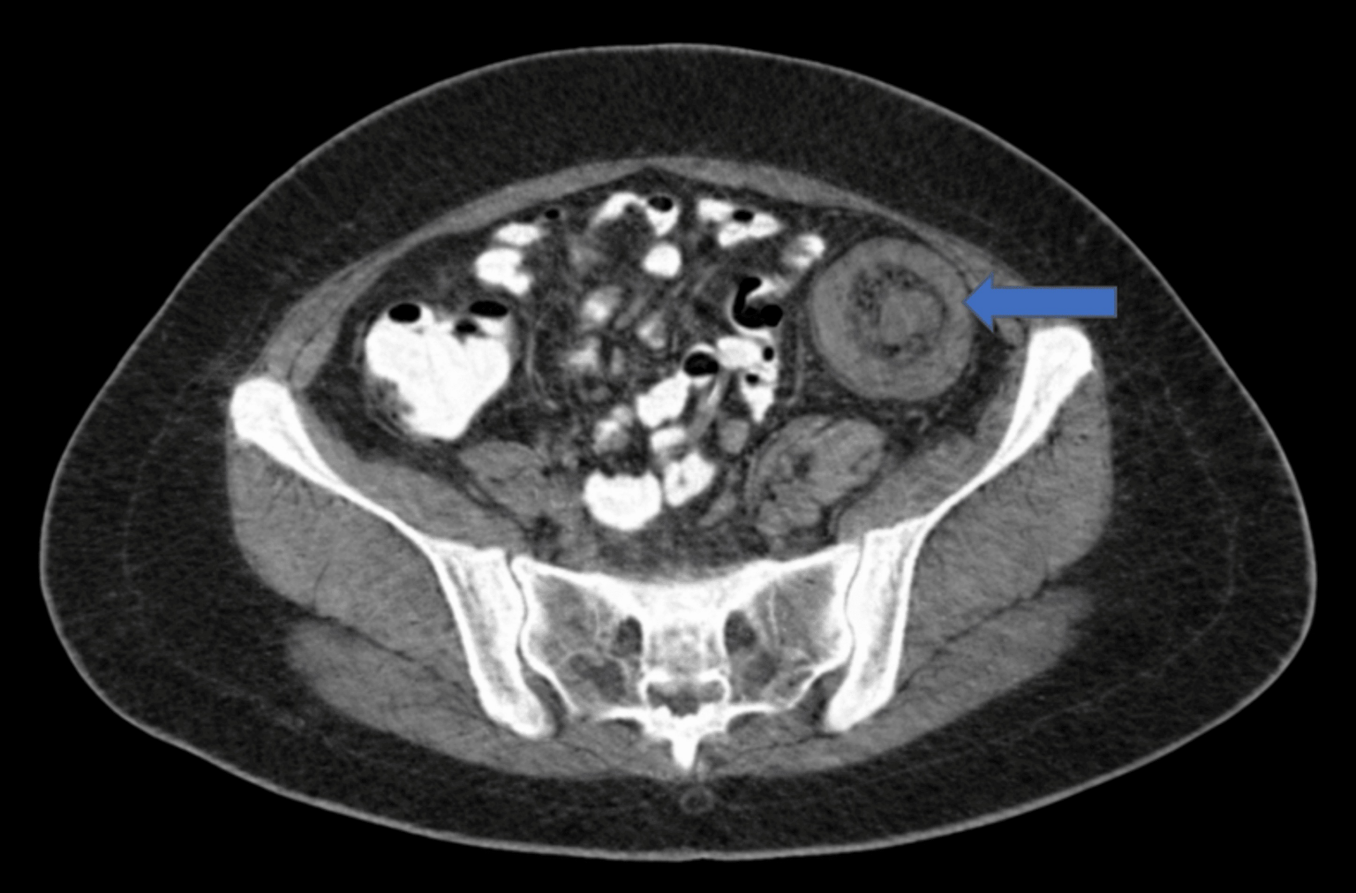
Concentric appearance on axial plane and tubular appearance on longitudinal plane, associated with striation of the adjacent peritoneal fat, suggestive of intussusception

A contrast-enhanced thoraco-abdomino-pelvic CT scan (Figure 3) demonstrated a well-defined intraluminal mass located at the descending colon with poorly defined parietal thickening; the lesion appeared submucosal in origin and exhibited predominantly fat-density attenuation, associated with striation of the adjacent peritoneal fat, suggestive of intussusception. Inflammatory changes in the peritoneal fat adjacent to the descending colon were observed.

On preoperative CT imaging, the lesion appeared to be located predominantly in the descending colon. However, intraoperatively, the lesion was identified in the sigmoid colon. 

The patient underwent a laparoscopic sigmoidectomy with resection of the involved colonic segment.

The patient was placed in the modified lithotomy position with Trendelenburg and right tilt. Pneumoperitoneum was established with a Hasson technique, and four laparoscopic trocars were placed under direct vision.

Initial exploration confirmed a lesion located in the sigmoid colon. The sigmoid colon was mobilized using a medial-to-lateral approach. The inferior mesenteric vessels supplying the sigmoid colon were identified and divided as appropriate, preserving adequate vascularization of the remaining colon. The sigmoid colon was resected with proximal and distal margins of approximately 5 cm from the lesion. The specimen was extracted through a protected mini-laparotomy. Restoration of bowel continuity was achieved through a primary end-to-end colorectal anastomosis using a circular stapling device. The surgery was completed successfully with no intraoperative complications.

**Figure 4:**
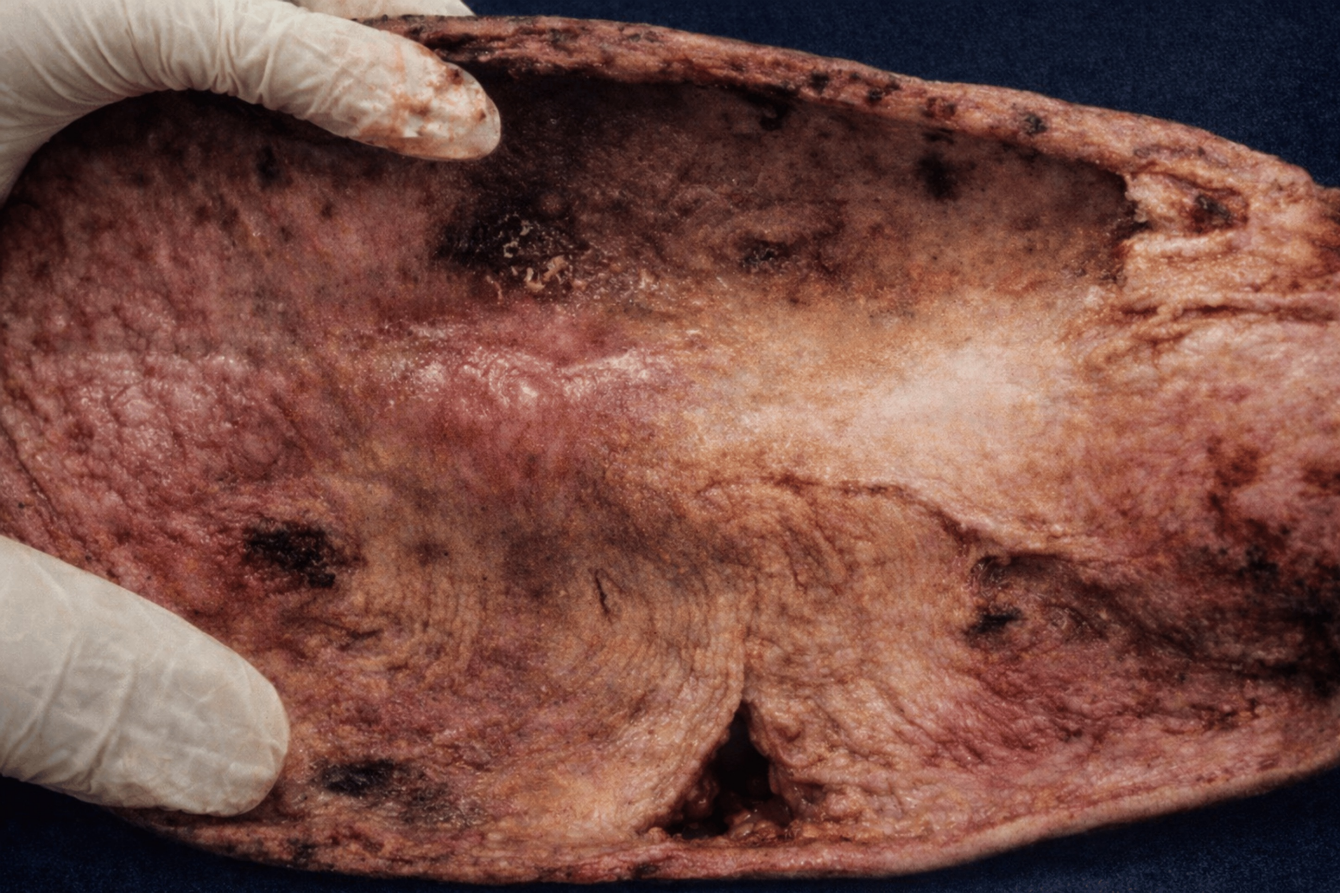
Giant sigmoid lipoma, measuring 8×5.8×4 cm with proximal and distal margins of 5 cm

Histopathological examination of the resected specimen confirmed a giant sigmoid lipoma, measuring 8×5.8×4 cm in greatest dimensions, with extensive transmural necrosis and ischemic changes in the mucosa, with the presence of vegetation; the surrounding colonic mucosa demonstrated chronic colitis with moderate inflammatory activity (Figure 4).

The patient had an uneventful postoperative course and was discharged after five days of hospitalization without complications. She was evaluated at follow-up visits on postoperative day 7 and one month after hospitalization, at which time she remained asymptomatic, with no abdominal pain, with clean, well-healed surgical wounds, and with normal bowel habits, passing stool and gas.

## Discussion

This case of a giant sigmoid lipoma (8 cm) illustrates how a benign lesion can mimic cancer when it appears ulcerated/necrotic and with an impression of intussusception.

Malignant transformation is exceedingly rare, and the main clinical challenge is distinguishing lipomas from malignant colonic lesions, especially when the overlying mucosa is ulcerated or hyperplastic [1,6,7]. Asymptomatic lipomas generally require no intervention, while symptomatic or large lesions may be managed with endoscopic resection or, in select cases, surgical excision, particularly if malignancy cannot be excluded or if there is associated intussusception or obstruction [1,3,5,6].

Colonic lipomas are most frequently asymptomatic, particularly when small (<2 cm), and are often discovered incidentally during imaging or endoscopy [3,7,8]. When symptomatic, clinical presentation is primarily determined by lesion size and location. The most common symptoms include abdominal pain, reported in up to 79% of symptomatic cases, and alterations in bowel habits such as constipation (up to 83% in some series), diarrhea, or obstructive symptoms [8,9,10,11]. Rectal bleeding is another notable presentation, occurring in approximately 16-23% of cases, and may be associated with anemia. Larger lipomas (>2 cm) are more likely to cause symptoms, including intussusception, which is rare in adults but can present with acute abdominal pain, vomiting, and signs of bowel obstruction. Other less frequent findings include weight loss, nausea, and, in rare cases, prolapse of the lipoma through the rectum, mimicking anorectal conditions such as hemorrhoids or rectal prolapse [12].

Physical examination may reveal abdominal tenderness or distension, but up to a quarter of patients may have a normal abdominal exam despite significant symptoms. The clinical challenge lies in differentiating colonic lipomas from malignant lesions, especially when presenting with bleeding or obstruction [5].

Colonic lipomas most commonly affect adults in the sixth to seventh decades of life, with mean or average ages at diagnosis consistently reported around 66-67 years [5,11].

The typical age range is between 40 and 70 years. Regarding sex distribution, the literature shows some variability: several studies report a slight female predominance (e.g., 64% female in one cohort and 57% female in a systematic review of intussusception cases), while others describe an approximately equal distribution between sexes [11].

A minority of reports suggest a male predominance, but these are less consistent and may reflect small sample sizes or case series. Overall, the most robust data support a predominance in older adults, with a slight female preponderance or near-equal sex distribution depending on the population studied [10]. 

When symptoms do occur, they are typically related to the size and location of the lipoma. The most common clinical presentations include constipation (reported in up to 83% of symptomatic cases) and abdominal pain (seen in 79-83% of cases); alterations in bowel habits, such as diarrhea, may also be present, though less frequently [5,11].

Physical examination may reveal abdominal tenderness or distension, but a significant proportion of patients may have a normal abdominal exam despite symptoms [5]. The clinical challenge is that colonic lipomas can mimic malignant lesions, especially when presenting with bleeding or obstruction, necessitating careful diagnostic evaluation [1,4,11].

The pathophysiological key is simple: the transmural necrosis results from ischemic compression of the overlying mucosa, not from oncological aggressiveness. On imaging, the fat signature suggests a lipoma, but this can be masked by edema/hemorrhage and volume effects; therefore, peripheral hydronephrosis measurements are advisable. The most effective imaging techniques for diagnosing colonic lipomas are CT, CT colonography (CTC), and endoscopic ultrasound (EUS). CT and CTC are highly sensitive for identifying colonic lipomas due to their characteristic low attenuation values, typically in the range of -41 to -258 Hounsfield units, which reflect the fatty composition of these lesions. This allows for confident differentiation from other colonic masses, including malignant lesions, and enables the detection of both sessile and pedunculated lipomas, often at smaller sizes than optical colonoscopy can achieve [4,9].

MRI can also demonstrate the fatty nature of lipomas, but CT remains the preferred modality for both diagnosis and preoperative planning due to its ability to exclude other pathologies and provide detailed anatomical information [4].

Endoscopic evaluation, including colonoscopy, can reveal typical features such as the "cushion sign" and "pillow sign," which are highly specific for lipomas, although not very sensitive. EUS further characterizes these lesions as homogeneous, well-defined, hyperechoic masses arising from the submucosal layer, and when these features are present, tissue sampling is generally unnecessary [2,7]. 

With this endoscopic-CT/pathological correlation, management is guided by caution: many lipomas are observed, some pedunculated lipomas are resolved by endoscopic resection, and large/complicated lipomas or those with oncological uncertainty are treated with segmental surgery [4,13].

Although imaging modalities are useful in aiding diagnosis, their findings are not conclusive; therefore, a definitive diagnosis must be made, and it is only established after the surgical excision of the lesion and histopathological confirmation of the diagnosis [13].

In summary, CT and CTC or virtual colonoscopy are the most effective imaging modalities for diagnosing colonic lipomas, with endoscopic and EUS features providing additional specificity when the diagnosis is uncertain [2,4,9,14].

## Conclusions

Giant colonic lipomas represent an uncommon cause of gastrointestinal symptoms and adult intussusception. The present case, supported by a review of the current literature, underscores the diagnostic value of cross-sectional imaging and endoscopy in distinguishing these lesions from malignant colorectal disease. Early and accurate diagnosis facilitates optimal treatment selection and prevents unnecessary radical surgical resections.
